# Literary Notes

**Published:** 1902-04

**Authors:** 


### LITERARY NOTES.

Dr. W. E. Fitch, founder, and for many years editor and business manager of *The Georgia Journal of Medicine and Surgery*, published at Savannah, Ga., has sold his interest in the publication to his former associate and co-editor, Dr. St. J. B. Graham, who becomes editor, and sole proprietor. The *Journal*, under Dr. Fitch's editorial management has merited the support of the profession, and gradually, year after year, has made for itself a place among the best medical periodicals of the period. Dr.

Fitch will devote his entire attention to the practice of his profession in Savannah.

---

A Treatise on smallpox, price $3.00, is announced for publication early in April by J. B. Lippincott Company. It is written by Dr. George Henry Fox, professor of dermatology in the College of Physicians and Surgeons, New York City, with the collaboration of Drs. S. Dana Hubbard, Sigmund Pollitzer, and John H. Huddleston, all of whom are officials of the health department of New York, and have had unusual opportunities for the study and treatment of this disease during the present epidemic.

The work is to be in atlas form, similar to Fox's Photographic Atlas of Skin Diseases, published by the same house. A strong feature of the work will be its illustrations, reproduced from recent photographs, the major portion of which will be so colored as to give a very faithful representation of typical cases of variola in the successive stages of the disease, also unusual phases of variola, vaccinia, varicella, and diseases with which smallpox is liable to be confounded. These illustrations number 37 and will be grouped into ten colored plates, $9\frac{1}{2}$ x $10\frac{1}{4}$ inches, and six black and white photographic plates.

The names of Dr. Fox and his associates assure the excellence of the work, in which will be described the symptoms, course of the disease, characteristic points of diagnosis, and most approved methods of treatment.

